# Expériences Relative Aux Effets De L’Inhalation De L’Ether Sulphurique Sur La Système Nerveux

**Published:** 1847-08

**Authors:** 


### REVIEWS.

---

ART. III.—*Expériences Relative aux Effets de l'Inhalation de l'Ether Sulphurique sur le Système Nerveux.* Par F. A. LONGET; Professeur d'Anatomie et de Physiologie, etc., etc. Mémoire lu à l'Académie Royale de Médecine. Victor Masson, Paris, 1847. pp. 54; 8vo.

*Experiments relative to the Effects of the Inhalation of Sulphuric Ether on the Nervous System.* Read to the Royal Academy of Medicine. By F. A. LONGET, &c., &c.

This publication, for which we are indebted to the kindness of our Paris correspondent, is the first systematic and sustained attempt that we have seen to explain the action of Sulphuric Ether—Letheon—on the animal economy. The author has an established reputation as a writer on the anatomy and physiology of the nervous system, and may be supposed to be peculiarly fitted for an investigation like the present. We have read this paper with a good deal of interest, and shall proceed to give a short account of it.

Longet first inquires whether animals submitted to the inhalation of ether had merely a concentration of the peripheric sensibility in the centres of the nervous system; or whether those portions of the cerebro-spinal axis, sensible to the ordinary means of excitation, lost their sensibility like the nervous cords themselves.

The following fact was already known to him. In an animal recently killed the motor principle forsakes first the encephalon, next the spinal marrow, then the nerves from the central towards the muscular extremities, or in a centrifugal direction; whilst the sensory principal is lost in a centripetal direction. In other words, sensibility first disappears in the smaller branches of the nerves, then in the larger, in the nerves themselves, in the posterior spinal roots (lumbar, dorsal, cervical) and gradually in the posterior bundles of the spinal marrow (lumbar, dorsal, cervical) ascending towards the encephalic centres.

The knowledge of this fact led the author to suppose that, in the peculiar ebriety produced by sulphuric ether, the parts of the cerebro-spinal axis ordinarily sensible might be so still, even though the nerves themselves might be absolutely insensible. But experiment did not bear him out; for this absolute insensibility was found *as well in all the central as in all the peripheral portions of the nervous system.* Even the electric current, the most energetic of all known stimulants, failed to reveal the existence of sensation in any part of the sensitive nervous apparatus. It is well known that, applied to the optic nerve of an animal at the point of death, it causes a luminous sensation indicated by motions of the iris; but in an animal under the influence of ether it does not produce this effect.

In the healthy state, according to the views of the author, the *great nervous centres* exhibit sensibility in the following points: the posterior portions of the varolian bridge and of the rachidian bulb, the quadrigeminal tubercles at a certain depth, and the posterior bundles of the spinal marrow. In the *peripheral nervous system*, the ganglionic portions of the trigeminal, glosso-pharyngeal, and pneumo-gastric nerves, and the posterior roots of the spinal nerves.

Inasmuch as the sensory principle follows an ascending or centripetal course in its progressive extinction, it is evident that it must abandon last the posterior portions of the varolian bridge and the rachidian bulb; at a certain period of the experiment it does in fact abandon them, *and yet the animal continues to breathe and to live, so as to recover subsequently all its faculties.* This persistence of respiration with a bulb in a state of anæsthesia is frequently met with by the pathologist. It is seen in the moribund and the apoplectic, where he bulb, no longer transmitting either sensitive impressions, or cerebral action on the voluntary muscles, still acts as the primary motor of the respiratory mechanism." The special seat of this respiratory influence, according to Longet, is not in the entire thickness of the bulb, but in the bundle intermediate between the pyramidal and the restiform bodies. These may be cut without affecting the respiration; but the destruction of the intermediate bundle alone, at the same level, instantaneously suspends that function. This intermediate portion is not exclusively formed of white fibres, like the remainder of the cord, but includes a considerable amount of greyish matter; and so long as this retains its integrity, the respiratory movements are maintained.

As to the *motor nervous apparatus*, though generally disordered and depressed in its action as shown by the muscular relaxation in man, it still responds to galvanic irritation in the etherized animal; and indeed the normal relations between muscular contraction and the direction of the electric current, are still present. The excitability of the anterior bundles of the spinal marrow, of the anterior spinal roots, and of the motor nerves of the cranium, in the etherized animal, may be seen also after death on applying the electric current; but it does not continue so long as in cases where death has has been produced from some other cause.

An animal then under the influence of ether only loses temporarily, in consequence of profound but transient modifications of its encephalon, the power of executing *spontaneous* movements; but it cannot be said that the inciting motor principle, the *motor nervous force* properly so called, is at any time completely absent from any portion whatever of the motor nervous apparatus.

Strychnine and the preparations of opium possess the singular property of exaggerating or increasing the excito-motory or reflex power of the medulla oblongata; ether, on the contrary, acts in a manner precisely the reverse, and rapidly suspends this *proper spinal action.* For example, winking, which follows direct stimulation of the mucous membrane of the eye, and which is seen in animals just before death, and even some instants after death, does not occur in such as are rendered insensible by the inhalation of ether; and the most energetic irritants applied to the mucous membrane of the pharynx, excites neither movements of deglutition nor concomitant closure of the glottis. The reflex action of the medulla oblongata is absolutely suspended.

The senisibility of the pharynx and glottis being suspended, difficulty may arise from the flow of blood into the air passages during operations in the throat and nasal cavities. The *possibility* of such an occurrence had already been suggested, and though a small number of such operations may have been performed without untoward results, there is nothing in them which can give security for the future. When the quantity of blood is small, the danger pointed out may be at its minimum, or even have no existence; for the epiglottis in the erect position forms on the anterior wall of the pharynx, when the latter is at rest, a projection sufficient to turn aside from the superior opening of the glottis a small quantity of liquid, by dividing the stream into two columns, and directing these into the two lateral fossæ of the posterior wall of the larynx. If the blood flows along the posterior or lateral wall of the pharynx, it may also avoid the superior opening of the respiratory organs. Still in an operation on the parts indicated, attended with a great flow of blood, the patient at the same time not being allowed to lie down, the danger would really be very great; for in his experiments on animals under the influence of ether it has frequently happened to the author to asphyxiate them almost instantly by pouring into the mouth, without precipitation, the same quantity of water that passed easily in animals of the same kind whose pharynx was acting normally.

There would be great rashness in giving a patient, completely under the influence of ether, a large quantity of water to drink.

In general the observer can determine in what order the central parts of the nervous system of animals are affected by the ether; and Longet thinks that we possess in it the means of *isolating the seat of general sensibility from that of intelligence and will.* In the animals (dogs and rabbits) which were the subjects of his experiments, he has distinguished two periods, viz:

In the first, the animal no longer able to sustain itself, falls upon its side, and is agitated, grows sleepy, and soon becoming unconscious of the external world, no longer executes any spontaneous movements, and remains plunged in a profound sleep; still, it throws itself about, and gives utterance to cries, when any sensible portion of the body is strongly pinched, but does not awake so as to offer any efficient resistance to this external violence. This is the *period of etherization of the cerebral lobes*, and even of the other parts of the encephalic mass, except the varolian bridge and rachidian bulb.

In the second, the animal having inhaled the ether for a longer time, no longer cries out, moves, or feels anything, even when the most sensitive portions of the nervous system are subjected to tractions and laceration. This is the *period of etherization of the varolian bridge*, the effects of which are associated with the preceding.

Now to prove that these variations in the phenomena are produced by the ether's affecting the designated portions of the brain:—If the encephalon of rabbits or dogs be mutilated *so as to leave only the varolian bridge and the bulb in the cavity of the cranium*, the animals, though apparently in a state of profound coma, will still, under severe external irritation, utter plaintive cries, and agitate themselves violently, *just as those who have undergone etherization of the cerebral lobes only.* But, if the varolian bridge is deeply injured, the moaning and agitation consequent on severe pinching immediately cease; there is left merely an animal in which the circulation, the respiration, and the other nutritive functions continue for a short time; and this animal, having lost its *perceptive centre of tactile impressions*, must, in a physiological point of view, be compared to that which has reached the *period of etherization of the varolian bridge*, or of absolute insensibility.

The foregoing observations are not without a practical bearing.

Certain patients, whilst undergoing operations, cry out violently, withdraw the limbs abruptly, and present the ordinary signs of suffering; but on coming to themselves, affirm that they do not know what has been done to them, that they recollect nothing, and have experienced no painful impression. The influence of the ether has been limited to the *lobes of the cerebrum*, and these persons are in a situation analogous to that of the animals deprived of the cerebral lobes but retaining still the annular protuberance, or the seat of perception of tactile impressions. Has the man, or the animal, in the cases referred to, had any real suffering? The author answers in the affirmative, and thinks that memory alone is wanting.

Assuredly, he says, there is no memory unless there has been perception; but memory is not necessarily present every time that a perception has existed. A man during sleep changes his position, turns back and forth without falling from his narrow couch, and tosses himself about violently if the integuments are stimulated; it is not to be supposed that he is absolutely deprived of sensations; and, because the perception of them has not been altogether distinct, because he does not recollect them, it does not follow that he has not had them. In the mid state between sleep and waking, how many ideas traverse our brain, and escape us the next moment?

Certainly, if the word *sensation* be taken in its strict metaphysical acceptation, and applied only to the exercise of sensibility with *consciousness*, it will be granted that the annular protuberance as the seat of sensibility, and the cerebral lobes as the seat of intelligence, must necessarily place their activity in common and concur to the same act.

But, strictly speaking, cannot the physiologist be allowed to distinguish the simple *perception* of tactile impressions, from the attention that is accorded to them, and the aptitude to form ideas in relation with them? Attention and the ulterior formation of ideas are subordinate to the participation of the cerebral lobes, the loss or etherization of which may bring on stupor, without abolishing the exercise of general sensibility, which is immediately subordinate to the varolian bridge. Admitting that the latter may, by itself (*isolèment*), act as the *centre of perceptivity*, the brain properly so called (the cerebral lobes) is nevertheless to be considered as the organ of essential elaboration, where tactile sensations in particular are appreciated, so to speak, at their just value, where they take a distinct form; as the organ consequently which is the seat of memory, the faculty by means of which it supplies the animal with the materials of its judgments and determinations, &c.

But the author furnishes some comparative experiments on this point. He laid bare the sciatic nerve in three animals, after having carefully concealed the remainder of their bodies from the sight of the observers. The three nerves were successively and repeatedly pinched, and each time there was great agitation, and outcries equally plaintive on the part of each of the animals. The unanimous opinion was, that suffering existed beyond doubt in each of the three cases. Now of these animals, the first was etherized to the first degree (*etherization of the cerebral lobes*); the second retained of its encephalon only the annular protuberance and the bulb; the third was altogether intact save the wound in the thigh. The second was subjected to further mutilation in the removal of the varolian bridge, and though it continued to live and breathe, it remained quiet, making no moan when the scalpel or the forceps was applied to the most sensitive regions. In the first the inhalation of ether was carried a little further, to the point of *etherization of the varolian bridge*, and the same absolute insensibility supervened. The varolian bridge is therefore indispensable to the exercise of general sensibility; it represents the primary perceptive centre of tactile impressions which are elaborated in another organ; and ether is a preventive of pain only on condition of its acting on this part of the brain.

It may be said that if patients lose the memory of pain it is quite the same as if they had not experienced any. But the assertion is not sustainable; for if it be admitted that the patients in the foregoing cases really suffered, the shock to the constitution must have been quite as great as if the operation had been performed under ordinary circumstances.

The physiological condition of the *great sympathetic* undergoes some modification in animals that have inhaled ether. The vermicular motions of the intestines immediately after death have appeared to the author to be less active, and of shorter duration, than in animals killed by section of the rachidian bulb. The pulsations of the heart have likewise appeared to be less energetic and of shorter duration. It must not be supposed, however, that these organic movements suffer abatement during life; for observation goes to show that their speedier cessation after death may depend on their temporary exaltation during the life of the animal. The pulse, in fact, is observed to increase in frequency during the first moment of an experiment, then it abates, though still above the healthy standard; if the experiment is prolonged so as to endanger life, the cardiac contractions again become hurried and more numerous. As to the intestines, it is not unusual, in dogs put to sleep by ether, to see slight alvine evacuations, which in this case cannot be attributed to fright, but doubtless depend on the increased contraction of the alimentary canal. In man, at the moment of waking, copious vomiting has been observed.

We find no experiments here as to the effects of ether on uterine contraction; but reference is made to a paper on this subject submitted to the Academy by Dubois. We believe some portions of this paper have found their way into our Journal, under the head of Selections. Dubois points out, among other things, the contraction of the abdominal muscles which persists along with uterine contraction when all the remaining voluntary muscles are in a state of relaxation.

Longet explains this by reference to the rachidian bulb, which plays such a part in this paper. It is the "first motor of the respiratory mechanism." Upon it depends the maintenance of the respiratory movements—the dilatation of the nostrils or of the mouth, the opening of the glottis, the elevation of the ribs and shoulders, the contraction of the diaphragm and *abdominal muscles*, but only as muscles concurring in the act of respiration. Now, straining, or *nisus*, in general, and that of labor in particular, is but a modification, a transient change of the respiratory act; it is a state in which the muscles of the ribs and shoulders, the diaphragm, and the abdominal muscles must contract with energy; in which, also, according to Isidore Bourdon and J. Cloquet, the glottis is spasmodically closed; and in which many other muscles contract by virtue of a synergie of action. In etherization, though volition is absent, yet under the influence of the bulb all the muscles concerned in respiration continue to act, and of course any *nisus* resulting from the contraction of these muscles, including the abdominal, may also be produced. This, though oftenest under the influence of the will, may take place without it; as is seen in a certain period of labor, and sometimes in lithotomy or lithotrity, where contraction of the uterus or the bladder irresistibly induces contraction of the abdominal muscles and the diaphragm.

The perineum, which, according to Dubois partakes of the general muscular relaxation in the parturient female, does not belong to the muscular apparatus of respiration; and in the involuntary *nisus* of labor, being passive, it is depressed under the weight of the abdominal viscera. But in voluntary *nisus*, or straining, the muscles of the perineum contract like many other muscles *not directly influenced by the respiratory nervous centres*, merely by virtue of that synergie to to which reference has already been made.

We have noticed a few of what seemed to us the most interesting portions of this memoir, but as many things have been passed over, we quote entire the following propositions in which Longet makes a summary of the whole:

1. In etherized animals there is an absolute and temporary suspension of sensibility, as well in all the ordinarily sensitive parts of the cerebro-spinal axis (*posterior portions of the varolian bridge, of the bulb, of the spinal marrow, &c.*) as in the nervous cords themselves (*nerves of the members, posterior spinal roots, trigeminal nerve, &c.*)

2. The relation which normally exists between the direction of the electric current and the muscular contractions due to this current—a relation made known by Matteucci and myself—remains in the motor nervous apparatus (*nerves of the members, anterior spinal roots, anterior cords of the spinal marrow, &c.*)

3. However, by means of galvanism, it is shown that after death the irritability of the muscles and the excitability of the nerves of motion do not continue so long in animals killed by ether as in those dead from some other cause, from section of the bulb for example.

4. Every mixed nerve (*sciatic, &c.*) which, exposed in a part of its course and subjected to the action of ether, has become insensible at the point directly etherized and at all those that are below it, may nevertheless continue *excitable* by galvanism at the same points, and, on certain conditions, may in part preserve its voluntary motor faculty.

5. Electrical or mechanical irritation of the optic nerve, which, even in an animal almost dead, causes a luminous sensation indicated by the motion of the pupils, does not produce the least effect in an animal rendered insensible by ether.

6. The action of ether on the sensitive nervous apparatus is far more direct and stupefying than that of alcohol, which merely renders sensibility more obtuse, without ever entirely suspending it, at least in the nervous centres.

7. Ether abolishes, temporarily but completely, the excitomotory or *reflex* property of the spinal marrow and the medulla oblongata (*proper spinal action*), and consequently acts inversely as strychnine and the preparations of opium, which exalt it.

8. In animals submitted to experiment, the effect of ether on the excito-motory function of the spinal marrow may be lessened or even neutralized by strychnine, and those of strychnine by ether.

9. The functions of the encephalic centres are constantly suspended before the proper spinal action, and are renewed before it.

10. Ether furnishes a new means of experimental analysis, which, employed with discretion, enables us to isolate, in a living animal, the seat of general sensibility from that of intelligence and of will.

11. The action of ether upon the nervous centres in living animals, may be regulated, and the two periods which I have called *period of etherization of the cerebral lobes*, and *period of etherization of the varolian bridge*, produced at will.

12. These two periods are easily reproduced by mutilations of the brain in living animals: in an animal which retains only the varolian bridge and the bulb, the phenomena correspond to etherization of the cerebral lobes ; in one where the varolian bridge itself has been directly injured, they correspond to etherization of this part.

13. Ether does not constitute a preventive of pain unless it acts on the varolian bridge.

14. In animals that have undergone etherization of the varolian bridge, this organ always recovers its function of perceptive centre of tactile impressions, before becoming again a sensible organ.

15. The progress of the phenomena of etherization is far from being rigorously the same in man as in animals.

16. The dis-etherization of the varolian bridge may begin, even whilst the influence is still felt by the cerebral lobes; which accounts for the cries uttered towards the end of an operation begun in a state of the utmost tranquillity—cries of which the patient retains no memory on waking.

17. The *true surgical period* corresponds to that of *etherization of the varolian bridge*, or of absolute insensibility.

18. A short time after the power of feeling has returned in etherized animals, there is a transient exaltation of sensibility.

19. Ammonia, either liquid or in the state of vapor, has appeared to me in a certain number of cases to shorten the duration of the phenomena arising from etherization; but only when these phenomena had not yet reached our second period.

20. At a certain period of the experiments, the blood, as first seen by Amussat, and subsequently by myself, becomes almost black in the arteries; *but insensibility invariably precedes this phenomenon.*

21. If the inspiration of ether, of the same quality, is continued after absolute insensibility occurs, the animals (rabbits) die in from six to twelve minutes, the temperature being from 6° to 8° Cent.

22. On the contrary, if there is a larger quantity of atmospheric air mixed with the ether, the period of absolute insensibility may be kept up for a very long time (three-quarters of an hour and more) without endangering the life of the animals (rabbits).

23. Ether, injected into the stomach through the œsophagus (even in sufficient quantity to prove fatal) does not cause loss of sensibility whilst vitality remains in the animal.

24. In etherization the functions of the ganglionic system of nerves seem to be sur-excited; this system apparently becoming a sort of *diverticulum* for the nervous force which has temporarily abandoned the cerebro-spinal system.

25. The death of animals that have inhaled too much ether is perhaps owing to a sort of asphyxia, the starting point of which would be especially in the respiratory nervous centre itself.

